# Overexpression of Notch Signaling Induces Hyperosteogeny in Zebrafish

**DOI:** 10.3390/ijms20153613

**Published:** 2019-07-24

**Authors:** Sung-Tzu Liang, Jung-Ren Chen, Jhih-Jie Tsai, Yu-Heng Lai, Chung-Der Hsiao

**Affiliations:** 1 Department of Bioscience Technology, Chung Yuan Christian University, Chung-Li 32023, Taiwan; 2 Department of Biological Science & Technology College of Medicine, I-Shou University, Kaohsiung 84001, Taiwan; 3 Department of Chemistry, Chinese Culture University, Taipei 11114, Taiwan; 4 Center for Biomedical Technology, Chung Yuan Christian University, Chung-Li 32023, Taiwan; 5 Center for Nanotechnology, Chung Yuan Christian University, Chung-Li 32023, Taiwan

**Keywords:** bone, hyperosteogeny, notch, transgenic zebrafish

## Abstract

Notch signaling is one of the evolutionarily conserved signaling pathways in multicellular organisms. It plays an important role in embryonic development. During skeletal development of vertebrates, it regulates bone homeostasis by manipulating both osteoblastogenesis and osteoclastogenesis through different mechanisms. However, due to the different nature of Notch signaling in mesenchymal stem cell and osteoblast, regulation of Notch signaling in bone-related diseases remains unsettled. Previous studies by cell culture and mouse models showed contradictory results regarding the role of Notch signaling in bone homeostasis. To clarify the role of Notch signaling in osteogenesis, we established a zebrafish model, in which Notch1a intracellular domain (N1aICD) was specifically expressed in the osteoblasts. We found that overexpression of N1aICD in osteoblasts caused hyperosteogeny in the column region of zebrafish with the morphology of narrowed neural/hemal canals. Moreover, increased metabolic activity of osteoblasts instead of augmenting osteoblast number led to hyperosteogeny in N1aICD-overexpressed zebrafish. In summary, we successfully established a transgenic zebrafish line overexpressing N1aICD to clarify the in-vivo function of Notch signaling during osteoblastogenesis. In the future, this fish line can serve as a valuable tool to test the therapeutic drugs for hyperosteogeny.

## 1. Introduction

Among myriad biochemical pathways, Notch signaling is one of the most conserved signaling cascades during skeletal development. Notch signaling is activated when the transmembrane ligands, such as Serrate/Jagged1 and 2; Delta Like1, 3, and 4; and contactin/F3/NB-3 [1,2,3] encounter Notch 1–4 receptors on the adjacent cells through cell–cell contact [4]. Canonical Notch signaling is triggered by a series of proteolytic cleavages, resulting in release of the ectodomain after A-Disintegrin-And-Metalloprotease (ADAM) cleavage [5], and translocation of the Notch intracellular domain (NICD) from the membrane to the nucleus, after cleavage by γ-secretase. Finally, NICD forms a complex with CSL (CBF1 in human, Suppressor of Hairless (Su(H)) in *Drosophila*, LAG1 in *C. elegans*; also called RBP-Jkappa in mice) DNA-binding protein and several coactivators (i.e., Mastermind/Lag-3, MAML) to activate downstream target genes on transmitting Notch signaling [6].

Two procedures are involved in bone formation: osteoblastic differentiation and osteoclastic inhibition. During osteoblastogenesis, mesenchymal stem cells (MSCs) are induced to differentiate into osteoblasts which can synthesize collagen and special proteins like osteocalcin as well as osteopontin to form the organic matrix and secrete hydroxylapatite deposited into the organic matrix to form a mineralized matrix. Previous studies showed diverse results in different experimental designs. In the past decade, in-vivo studies have shown Notch signaling plays an important role in regulating osteogenesis. Active Notch signaling maintains MSCs in an active proliferation state through persistent activation of the TNF pathway, which continuously promotes NICD translocation onto *Hes1* promoter to inhibit osteoblast differentiation and bone formation in a mice rheumatoid arthritis model [7]. Moreover, immature and dysfunctional osteoblasts were observed in NICD overexpressed transgenic mice with retardation of bone volume induction and growth [8]. In addition, previous studies showed that NICD activation at different developmental stages might prevent osteoblasts from differentiation through two possible mechanisms: either osteoblasts formed but failed to differentiate, or osteoblast development was arrested at an early stage so that there was a decreasing number of mature osteoblasts [9]. Studies have shown that Notch signaling served both stimulatory and inhibitory effects on osteoclastogenesis [10]. Loss of Notch 1 and 3 decreased the osteoprotegerin/RANKL expression ratio and induced proliferation of osteoclasts [11]. This suggested Notch suppresses osteoclastogenesis and, therefore, suppresses bone remodeling [12]. However, Notch 2 has been reported as an osteoclastogenesis remodeling stimulator, which regulates the promoter of a nuclear factor of activated T cells c1 (NFAT-c1) during osteoclast differentiation and induces osteoclastogenesis [13]. Taken together, Notch signaling plays an indispensable role in skeletal development and regulates differentiation of osteoblasts and osteoclasts. However, previous studies showed conflicting results depending on experimental conditions.

Zebrafish has been well known as a great animal model for studying development and diseases of vertebrates. Therefore, in this study, we aimed to clarify the role of Notch signaling in bone development using zebrafish model. To this end, we generated the zebrafish line Tg(Ola.Sp7:N1aICD)^cy31^ specifically over-activating the Notch intracellular domain (N1aICD) under the control of *sp7* promoter in osteoblasts, and a control zebrafish line Tg(Ola.Sp7:EGFP)^cy25^ that expressed EGFP driven by the *sp7* promoter. The *sp7* gene encodes a zinc finger-containing transcriptional factor expressed in osteoblasts so that it can be a marker of osteoblasts [14]. The medaka-derived *sp7* promoter has been widely used in generating transgenic models. With these zebrafish transgenic lines, we were able to investigate the effect of N1aICD overexpression on the embryonic skeletal development by evaluating the mineralization level with calcein staining and quantifying the area of ring centrum formation in zebrafish embryos at different developmental stages. We also evaluated the effect of N1aICD on bone development at late developmental stage by evaluating their body length, bone density and morphology. To clarify the mechanism of N1aICD-assoaciated hyperosteogeny, we monitored proliferation and differentiation of osteoblasts by quantifying osteoblast number and osteoblast activity as well as osteoclast marker gene expression at both protein and mRNA levels.

## 2. Result

### 2.1. Generation of the Transgenic Fish Line Tg(Ola.Sp7:N1aICD)^cy31^

**Figure 1:**
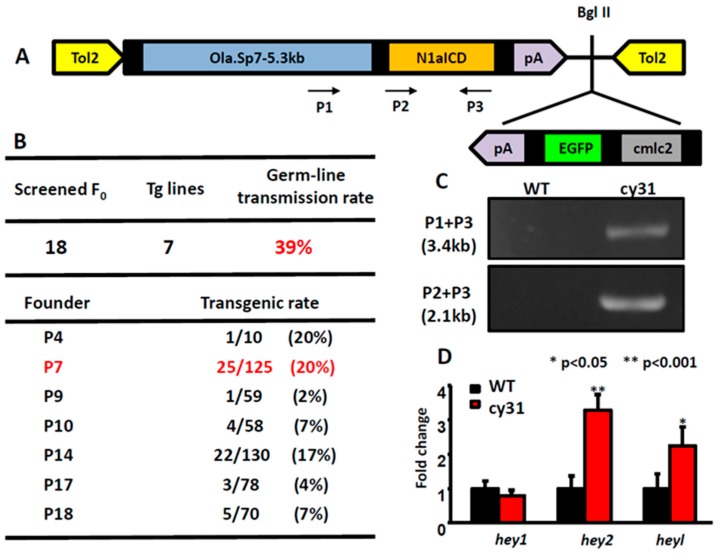
Generation of Tg(Ola.Sp7:N1aICD)^cy31^ transgenic fish. (**A**) Schematic diagram of vector (pDestTol2CG2-Ola.Sp7-N1aICD-pA), in which *notch1a*ICD (N1aICD) was driven by the medaka fish osteoblast specific promoter Sp7 and linked with *EGFP*, driven by *cmlc2*, a heart-specific promoter. (**B**) Genotyping to confirm the transgenic rate in different founder fish. Founder fish number 7 (P7) with high germ-line transmission rate was used to generate F1 progeny (labeled with red color). (**C**) Agarose gel electrophoresis validated N1aICD construction in the transgenic fish line Tg(Ola.Sp7:N1aICD)^cy31^. Genomic DNA from caudal fin was used as the template. PCR product was expected to have a size of 3.4 kb or 2.1 kb with primer P3 paired with P1 or P2, respectively. (**D**) Significant up-regulation of *hey2* and *heyl* expressions in Tg(Ola.Sp7:N1aICD)^cy31^ transgenic fish was observed by real time RT-PCR. (averages ± SD; * *p* < 0.05; ** *p* < 0.001; *** *p* < 0.005; n = 10).

**Figure A1:**
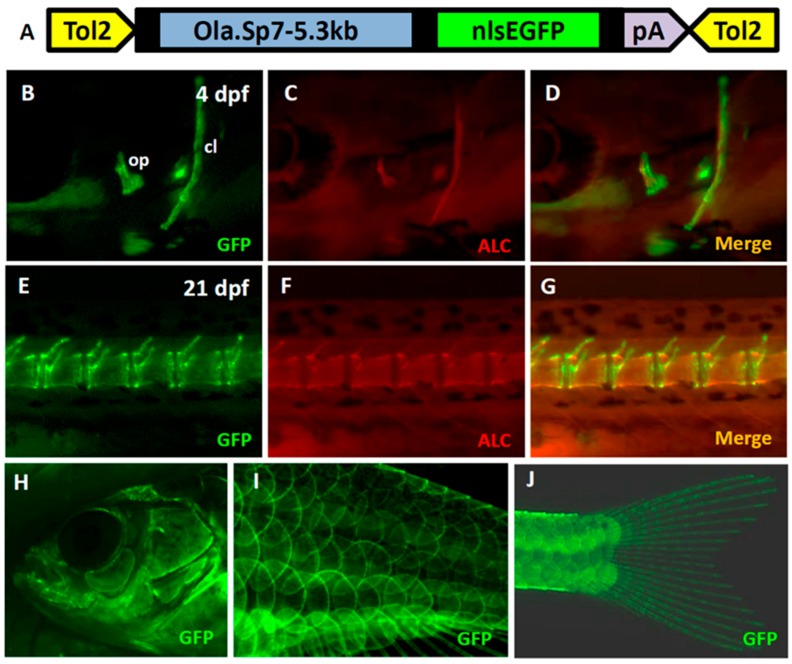
Generation of Tg(Ola.Sp7:EGFP)^cy25^ as a control fish to show the osteoblast-specific property of *sp7* promoter. (**A**) Schematic diagram of vector (pDestTol2CG2-Ola.Sp7-nlsEGFP-pA) which was used to express EGFP reporter gene driven by a 5.3kb Medaka *sp7* promoter uniquely in osteoblasts. Green fluorescence was expressed in opercula and cleithrum (**B**), notochord (**E**), fish head (**H**), body scales (**I**) and caudal fin (**J**). The transgenic fish was also counterstained with alizarin complexone (ALC) for mineralized tissue staining (red fluorescent color). The ALC stained opercula and cleithrum as well as notochord were shown in (**C**,**F**), respectively. The merged images for EGFP (green) and ALC (red) in opercula and cleithrum and notochord were highlighted in (**D**,**G**), respectively.

In order to create zebrafish lines specifically expressing Notch signaling in osteoblasts, we constructed a Tol2-based gateway vector which harbors the intracellular domain sequence of *notch1a* (N1aICD) driven by an osteoblast-specific *sp7* promoter (Figure 1A). Inside this vector, it also has a mini cassette, carrying the *EGFP* reporter gene driven by a heart-specific *cmlc2* promoter, to facilitate transgenic line screening. After microinjecting the in-vitro transcribed transposase mRNA and the Tol2-*sp7*-*notch1a*ICD construct into the fertilized eggs during the one-cell stage, we screened the three dpf embryos for EGFP expression in the heart as an indirect indicator to show whether embryo harbored the exogenous transgene. After outcrossing, we obtained seven independent transgenic founders that can successfully transmit the transgene through germline at different percentages. The germline transmission rate was calculated to be 39% (Figure 1B). Among all seven founders, we kept founder number 7 for further phenotype analysis and assigned it as Tg(Ola.Sp7:N1aICD)^cy31^ according to ZFIN nomenclature protocol. In addition, we also generated a control zebrafish line Tg(Ola.Sp7:EGFP)^cy25^ that expressed EGFP driven by medaka *sp7* promoter (Figure A1A). The transgenic fish line Tg(Ola.Sp7:EGFP)^cy25^ showed stably expressed EGFP in bone-related tissues, such as bones and scales where osteoblasts exist (Figure A1B,E,H,I,J). Next, we performed DNA genotyping to confirm the stable transmission of the transgene. Total genomic DNA isolated from the caudal fin was amplified with different primer pairs (indicated in P1, P2 and P3 in Figure 1A). These primers span the cDNA region of the N1aICD; therefore, the endogenous *notch1a* (spanning several exons and introns) cannot be amplified by these primers. The PCR-amplified product was resolved in agarose gel and showed the expected size (Figure 1C). To provide evidences to support exogenous N1aICD overexpression can activate endogenous Notch signaling in zebrafish, we measured the relative expression levels of well-known Notch downstream targets like *hey1*, *hey2* and *heyl* by RT-PCR for embryos aged at 3 dpf. The results showed that *hey2* and *heyl* were significantly upregulated in the Tg(Ola.Sp7:N1aICD)^cy31^ compared to WT (** *p* < 0.001 and * *p* < 0.05, respectively). However, the expression level of *hey1* showed no difference between wild type (WT) and Tg(Ola.Sp7:N1aICD)^cy31^, suggesting that *hey1* may not respond to N1aICD overexpression in zebrafish.

### 2.2. Calcification Level Increases in Early Embryonic Stage When N1aICD Overexpressed

**Figure 2:**
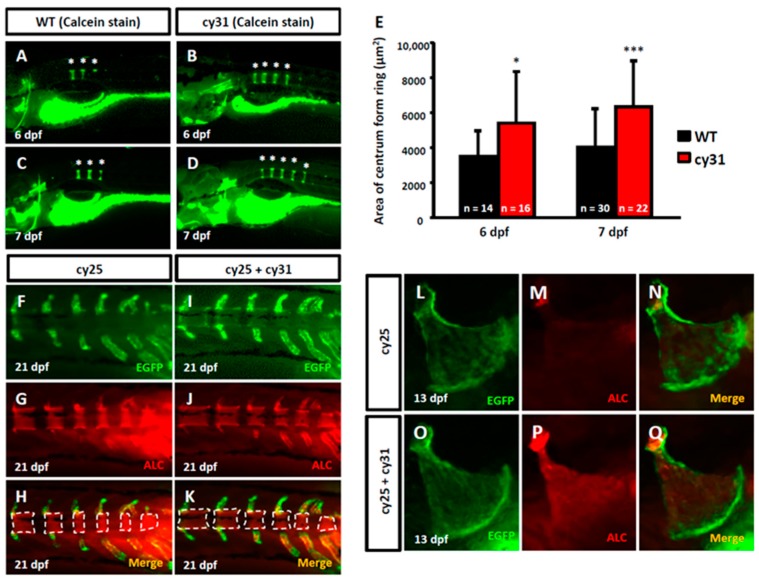
Evaluation of calcification in N1aICD-expressed transgenic zebrafish. (**A**–**D**) Calcein staining on wild type and Tg(Ola.Sp7:N1aICD)^cy31^ embryos at 7 days post-fertilization (dpf). The * indicates the formed column. (**E**) Quantification of calcification degree by calculating the area of ring centrum formation in the notochord. N represents the fish number used for analysis. (**F**,**G**,**H**) EGFP-expressed osteoblasts in green fluorescence, alizarin complexone (ALC) staining region and merged image in notochord of Tg(Ola.Sp7:EGFP)^cy25^ transgenic fish aged at 21 dpf. (**I**,**J**,**K**) N1aICD-expressed osteoblasts in green fluorescence, ALC staining region and merged image in notochord of double transgenic fish of Tg(Ola.Sp7:EGFP)^cy25^ and Tg(Ola.Sp7:N1aICD)^cy31^ at 21 dpf. The dashed line surrounded areas were measured using image quantitation and summed up for comparison. (**L**,**M**,**N**) EGFP-expressed osteoblasts in green fluorescence, ALC staining region and merged image in opercula of Tg(Ola.Sp7:EGFP)^cy25^ at 13 dpf. (**O**,**P**,**Q**) N1aICD-expressed osteoblasts in green fluorescence, ALC staining region and merged image in opercula of Tg(Ola.Sp7:EGFP)^cy25^ and Tg(Ola.Sp7:N1aICD)^cy31^ double transgenic fish at 13 dpf (averages ± SD; * *p* < 0.05; *** *p* < 0.005; n = fish number).

To investigate the effect of N1aICD overexpression on the embryonic skeletal development, we first evaluated the calcification level of zebrafish embryos aged at 6-7 dpf by calcein staining. We found the number of calcified vertebrate column significantly increased in Tg(Ola.Sp7:N1aICD)^cy31^ (Figure 2B,D) compared to WT (Figure 2A,C). The quantification results of the area of ring centrum formation were demonstrated in Figure 2E. At 21 dpf, the EGFP-positive osteoblasts appeared at the notochord area (Figure 2F,I), and the alizarin complexone (ALC)-positive notochord calcification was also detected (Figure 2G,J). The merged images demonstrated that the degree of calcification of notochord in double transgenic fish of Tg(Ola.Sp7:EGFP)^cy25^ and Tg(Ola.Sp7:N1aICD)^cy31^ (Figure 2K) is much higher than in Tg(Ola.Sp7:EGFP)^cy25^ control fish (Figure 2H). Similarly, the degree of calcification of opercula in the double transgenic fish of Tg(Ola.Sp7:EGFP)^cy25^ and Tg(Ola.Sp7:N1aICD)^cy31^ (Figure 2P) was significantly higher than that in Tg(Ola.Sp7:EGFP)^cy25^ with normal Notch expression (Figure 2M). These data suggested that overexpressed N1aICD may lead to increased calcification of bone during early embryonic development.

### 2.3. Hyperosteogeny Occurs in Late Adult Stage of N1aICD Overexpressed Zebrafish

**Figure 3:**
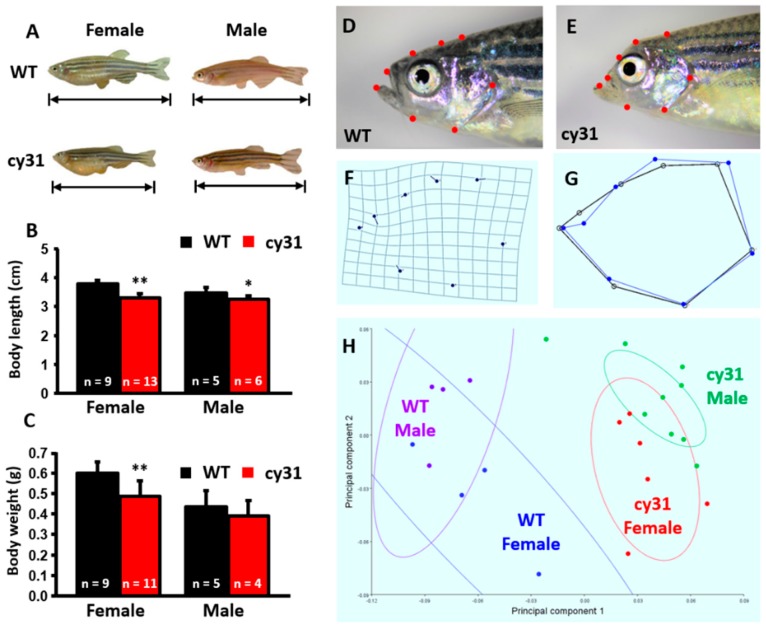
Morphological comparison between wild type and Tg(Ola.Sp7:N1aICD)^cy31^ adult fish. (**A**) Appearance of WT and Tg(Ola.Sp7:N1aICD)^cy31^ (cy31) transgenic fish in both genders aged 6 month old. (**B**,**C**) Statistical analysis and comparison of male/female body length (**B**) and body weight (**C**). (**D**–**G**) Morphometric analysis of the head in both WT and cy31 transgenic fish. (**H**) Principal component analysis (PCA) of WT and cy31 transgenic fish in both genders. Significance was tested by Student’s *t*-test and data were presented as averages ± SD (* *p* < 0.05; ** *p* < 0.01; n = fish number).

Generally, the overexpression of N1aICD in osteoblasts is not lethal to zebrafish embryos. Therefore, we were able to evaluate the effect of N1aICD on the bone development at late developmental stage. In the adult stage, body length is obviously significantly shorter in Tg(Ola.Sp7:N1aICD)^cy31^ than in WT for both genders (Figure 3A,B); however, the body weight was less only in females (Figure 3C). By morphometric analysis, we found significant head skull deformation in Tg(Ola.Sp7:N1aICD)^cy31^ (Figure 3E and blue line in 3G) when compared to WT (Figure 3D and black line in 3G). By principal component analysis (PCA), we found that the skull morphometric data between WT and Tg(Ola.Sp7:N1aICD)^cy31^ show significant differences (Figure 3H). The skull outlook in Tg(Ola.Sp7:N1aICD)^cy31^ is collapsed.

**Figure 4:**
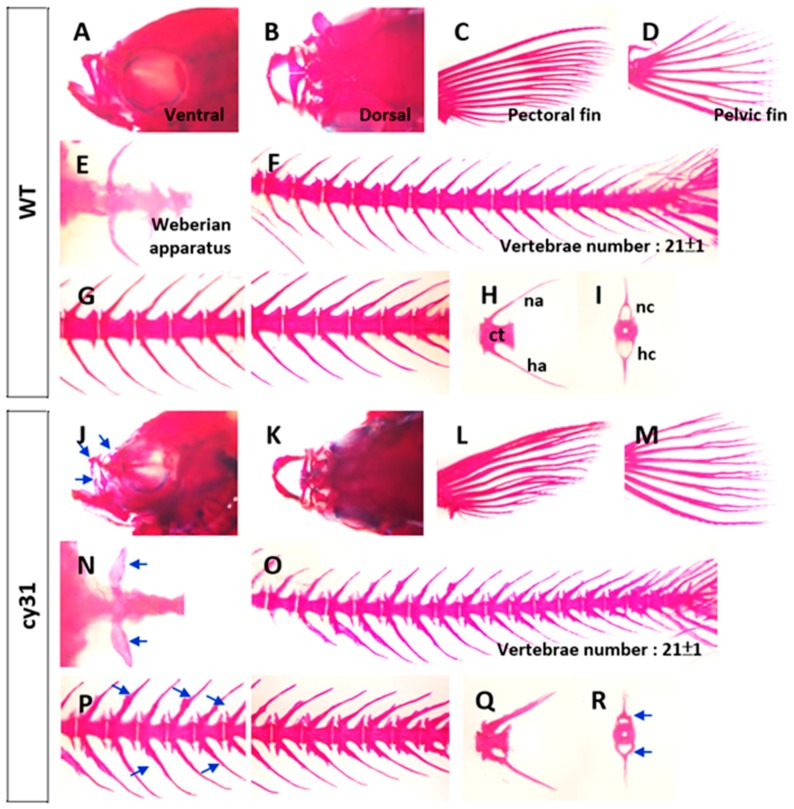
Alcian blue/Alizarin red double staining in different skeletal regions of wild type fish (**A**–**I**) and Tg(Ola.Sp7:N1aICD)^cy31^ transgenic fish (**J**–**R**). Bone morphologies of maxilla from lateral (**A**,**J**) and dorsal views (**B**,**K**), pectoral fin (**C**,**L**), pelvic fin (**D**,**M**), Weberian apparatus (**E**,**N**), vertebrae (**F**,**O**), and neural and hemal arches from lateral (**H**,**Q**) and anterior (**I**,**R**) views were magnified respectively. Blue arrows indicated the abnormal formation of outgrowing knots (centrum, ct; neural arch, na; hemal arch, ha; neural canal, nc; hemal canal, hc).

**Figure 5:**
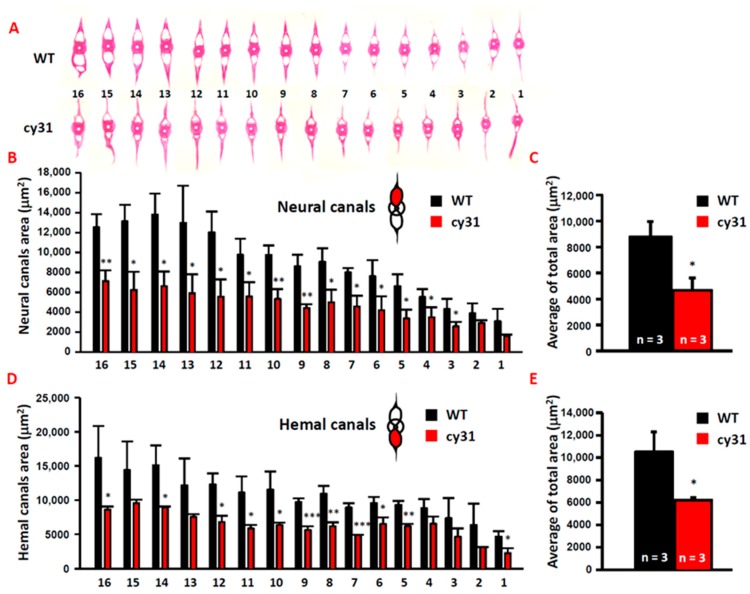
Overexpressed Notch causes narrow neural/hemal canals in Tg(Ola.Sp7:N1aICD)^cy31^. (**A**) Morphology of 16 sections of spinal bone in wild type and Tg(Ola.Sp7:N1aICD)^cy31^ transgenic fish (left to right represents the anterior to posterior positions). (**B**,**D**) Calculation of the size for neural/hemal canal cavities in each section of spinal bone in wild type and Tg(Ola.Sp7:N1aICD)^cy31^ transgenic fish. (**C**,**E**) Sum of the size in 16 sections of neural/hemal canal in wild type and Tg(Ola.Sp7:N1aICD)^cy31^ transgenic fish. Significance was tested by Student’s *t*-test and data were presented as averages ± SD (* *p* < 0.05, ** *p* < 0.01, *** *p* < 0.005; n = fish number).

To compare cartilage and bone morphology, inner skeleton was double stained with Alcian blue/Alizarin red. Consistent with morphometric analysis, we found that the Tg(Ola.Sp7:N1aICD)^cy31^ (Figure 4J,K) has a narrower and smaller head bone compared to WT (Figure 4A,B). The pectoral and pelvic fins of the Tg(Ola.Sp7:N1aICD)^cy31^ curved at the end (Figure 4L,M) instead of being smooth (Figure 4C,D). Moreover, the Weberian apparatus bone was thicker (Figure 4N), the neural and hemal arches in the spinal bone showed outgrowing knots (Figure 4O,P), and the arches were forked at the end (Figure 4Q) in Tg(Ola.Sp7:N1aICD)^cy31^ compared to WT (Figure 4C–H). Furthermore, the neural and hemal arches were thicker and the neural and hemal canals were narrower (Figure 4R) in Tg(Ola.Sp7:N1aICD)^cy31^. To quantify each size of neural and hemal canals, we dissected the spinal bones into 16 sections from tail to head. The area of the neural and hemal canal in each section was measured and compared in parallel between WT (Figure 5A upper panel) and Tg(Ola.Sp7:N1aICD)^cy31^ (Figure 5A lower panel). The inner capacity of each section of neural (Figure 5B) or hemal canal (Figure 5D) significantly decreased due to bone malformation. The total area of neural and hemal canals in all sections was also quantified, summed up and compared (Figure 5C,E). Results support the idea that N1aICD overexpression causes hyperosteogeny leading to the thickening of neural and hemal arches as well as narrow neural and hemal canal cavity in Tg(Ola.Sp7:N1aICD)^cy31^.

### 2.4. Notch Overexpression Causes High Bone Density and Swimming Behavior Alteration in Zebrafish

**Figure 6:**
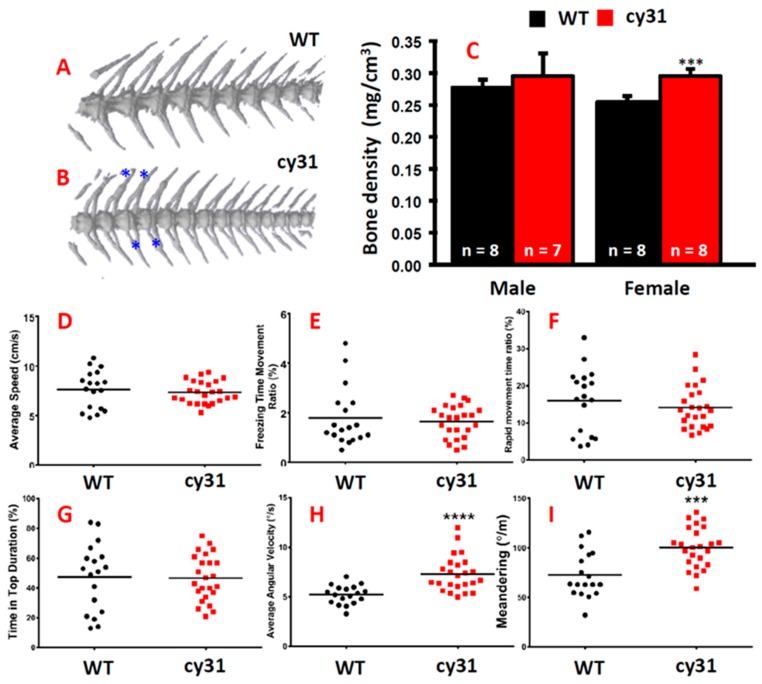
Computed tomography of both genders in wild type and Tg(Ola.Sp7:N1aICD)^cy31^ fish. (**A**,**B**) Three dimensional computed tomography for female WT, female Tg(Ola.Sp7:N1aICD)^cy31^, male WT, and male Tg(Ola.Sp7:N1aICD)^cy31^ fish. Three-dimensional computed tomography of spinal bone for WT and Tg(Ola.Sp7:N1aICD)^cy31^ fish. The blue * indicated the region where the knots occured (n = fish number). (**C**) Bone density of WT and N1aICD-overexpressing transgenic fish in both genders. (**D**–**I**) Comparison of three-dimensional locomotion swimming patterns between WT and Tg(Ola.Sp7:N1aICD)^cy31^ transgenic fish which are based on endpoints of average speed (**D**), freezing time movement ratio (**E**), rapid movement time ratio (**F**), time in tope duration (**G**), average angular velocity (**H**), and meandering (**I**). Significance was tested by Student’s *t*-test and data were presented as averages ± SD (*** *p* < 0.005, **** *p* < 0.001). Black circles and red squares represent the data for wild type and Tg(Ola.Sp7:N1aICD)^cy31^ fish, respectively.

The hyperosteogeny phenotype in Tg(Ola.Sp7:N1aICD)^cy31^ prompted us to ask whether the bone density also increased when N1aICD overexpressed in osteoblasts. Three-dimension micro computed tomography (µCT) was applied to scan WT (Figure 6A) and Tg(Ola.Sp7:N1aICD)^cy31^ fish (Figure 6B) at adult stage. Results showed the average bone density in female Tg(Ola.Sp7:N1aICD)^cy31^ was significantly higher than WT female; however, there was no difference among male fish (Figure 6C). These data suggested that female fish might be more sensitive to the N1aICD overexpression in inducing high bone density phenotype. In addition, we were interested to explore whether the hyperosteogeny phenotype causes any possible swimming behavioral alterations. To probe this question, we put 5–6 fish in a water tank and their 3D shoaling motion was captured by video recording, and locomotion trajectory was analyzed and compared according to our previous protocol [15]. The rationale to measure the swimming behaviors of zebrafish was to examine whether the hyperosteogeny phenotype affects the swimming patterns, such as speed, location preference, angular velocity, and meandering. In our previous studies, we have demonstrated that fish with anxiety showed increased swimming speed and tent to stay at the bottom of the tank. We did not observe any anxious swimming patterns in Tg(Ola.Sp7:N1aICD)^cy31^ with the hyperosteogenesis phenotype (Figure 6D–G). However, significant increased angular velocity in N1aICD-transgenic fish suggested that the increased bone density might augment the rotation of body and lead to the high speed of angular velocity (Figure 6H). In addition, the increased meandering was observed, which inferred the hyperosteogeny did not cause uncomfortability in fish (Figure 6I).

### 2.5. Increase of Osteoblast Activity Causes Hyperosteogeny in N1aICD-Overexpressed Zebrafish

**Figure 7:**
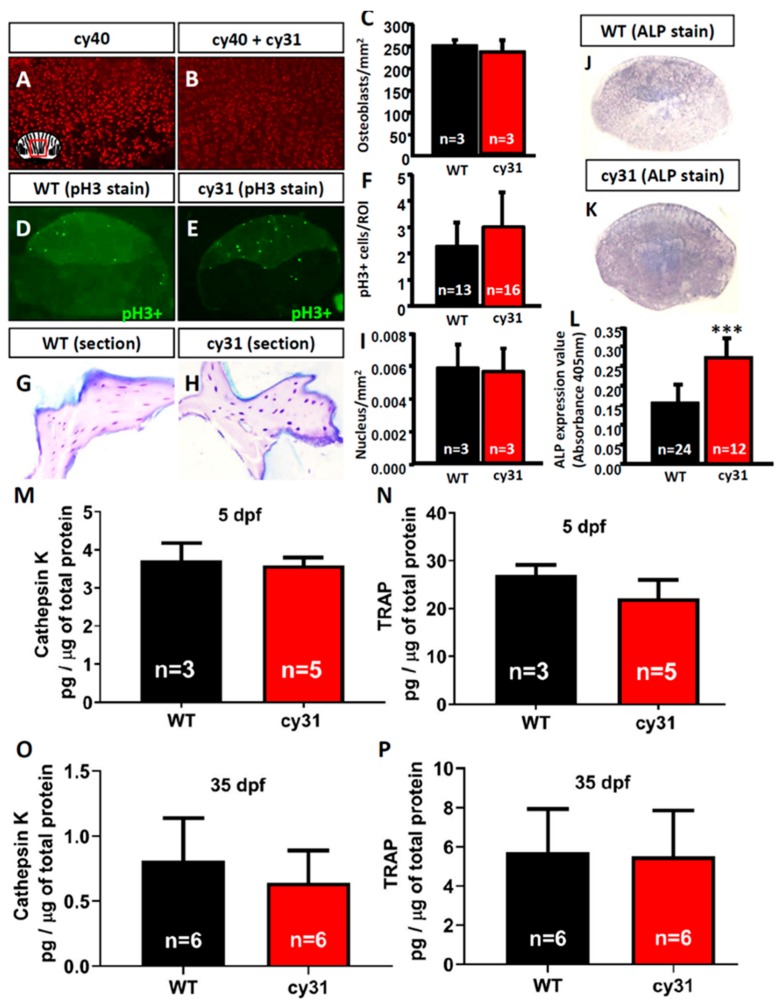
Detection of osteoblast and osteoclast activity in Tg(Ola.Sp7:N1aICD)^cy31^ fish. Quantification of the osteoblast number in wild type (**A**) and N1aICD-overexpressing Tg(Ola.Sp7:N1aICD)^cy31^ fish (**B**) scales with the aid of nucleus-tagged red fluorescent signals from Tg(Ola.Sp7:HA2FZmcherry)^cy40^. The quantitative and statistic comparison data for osteoblast number were summarized in (**C**). Phospho-Histone H3 (pH3) immunostaining to measure the mitotic division activity for wild type (**D**) and N1aICD-overexpressing Tg(Ola.Sp7:N1aICD)^cy31^ fish (**E**) scales. The quantitative and statistic comparison data for pH3-positive cell number were summarized in (**F**). H&E staining and nucleus quantitation of spinal bone sections for wild type (**G**) and N1aICD-overexpressing Tg(Ola.Sp7:N1aICD)^cy31^ fish (**H**). All number was determined by taking the average of osteoblast number in three areas on each individual scale or bone section. The quantitative and statistic comparison data for nucleus number were summarized in (**I**). Alkaline phosphatase (ALP) staining of scales in wild type (**J**) and N1aICD-overexpressed Tg(Ola.Sp7:N1aICD)^cy31^ fish (**K**). Statistical analysis of ALP-positive staining area in fish scales (**L**). Relative ALP activity in fish scale lysates was quantitatively detected at 405 nm. Quantitative comparison of cathepsin K (**M**,**O**) and TRAP (**N**,**P**) protein expression levels in wild type and N1aICD-overexpressed Tg(Ola.Sp7:N1aICD)^cy31^ fish aged at either 5 days poste-fertilization (dpf) (**M**,**N**) or 35 dpf (**O**,**P**). Significance was tested by Student’s *t*-test and data were presented as averages ± SD (*** *p* < 0.005; n = fish number).

**Figure 8:**
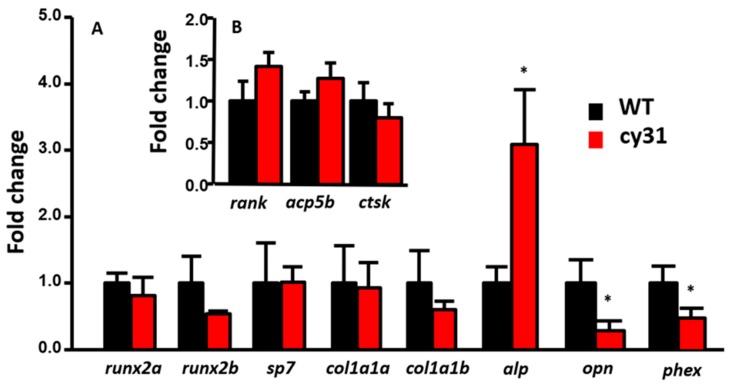
Relative expression levels of osteoblast downstream targeting genes and osteoclast-related genes in wild type compared to Tg(Ola.Sp7:N1aICD)^cy31^ fish. Total RNA was extracted from fish fin, and marker genes were tested including *runx2a*, *runx2b*, *sp7*, *col1a1a*, *col1a1b*, *alp*, *opn* and *phex* related to osteoblast activity (**A**); and *rank*, *acp5b* and *ctsk* related to osteoclast activity (**B**). Significance was tested by Student’s *t*-test and data were presented as averages ± SD (* *p* < 0.05; n = 10).

**Figure 9:**
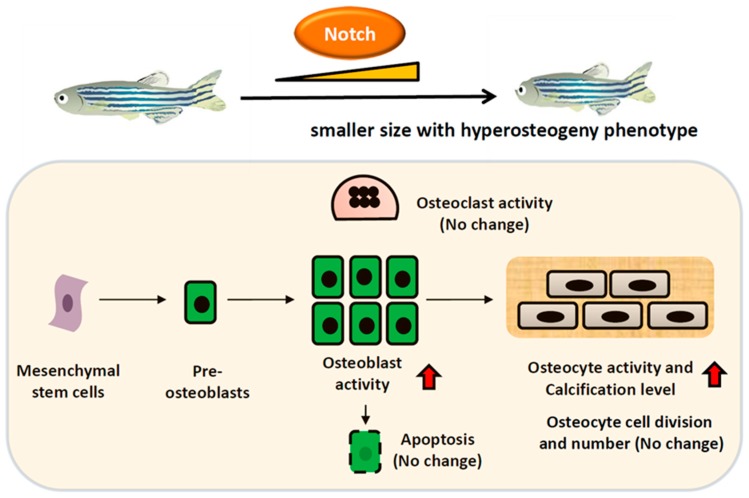
Schematic presentation of the effect of Notch activation on osteoblast. Increasing Notch expression results in the activation of osteoblasts which may differentiate into osteocytes that are embedded in the mineralized matrix to enhance bone density. On the contrary, the osteocyte number and osteoclast activity didn’t display significant alteration in Tg(Ola.Sp7:N1aICD)^cy31^.

To clarify the mechanism of N1aICD-associated hyperosteogeny, we monitored proliferation and differentiation of osteoblasts by quantifying osteoblast number and osteoblast activity. Because of the co-existence of osteoblasts and osteoclasts, fish scales have been used as a good model to monitor osteoblasts and osteoclasts activities ex vivo in several previous studies [16,17,18]. To count the osteoblast number, we first crossed the reporter line Tg (Ola.Sp7:HA2FZmcherry)^cy40^ with Tg(Ola.Sp7:N1aICD)^cy31^ to generate double transgenic fish. Result showed the relative osteoblast number (counted by red fluorescent in cell nucleus) in scales had no difference between Tg(Ola.Sp7: HA2FZmcherry)^cy40^ (Figure 7A) and double transgenic fish of Tg (Ola.Sp7:HA2FZmcherry)^cy40^ and Tg(Ola.Sp7:N1aICD)^cy31^ (Figure 7B). Moreover, neither phospho-Histone 3 staining on scales (Figure 7D–F) nor H&E staining on spinal bone sections (Figure 7G–I) in both WT and Tg(Ola.Sp7:N1aICD)^cy31^ showed a significant difference on osteoblast number (Figure 7I), indicating that hyperosteogeny may not be caused by the increase of osteoblast number. Later, we checked the osteoblast activity by measuring its alkaline phosphatase (ALP) activity. Results showed Tg(Ola.Sp7:N1aICD)^cy31^ (Figure 7K,L) expressed more ALP activity on scales than WT (Figure 7J,L), suggesting their osteoblasts are significantly more active. Next, we also validated the osteoclast activities in Tg(Ola.Sp7:N1aICD)^cy31^ by measuring the expression of cathepsin K and Tartrate-resistant acid phosphatase (TRAP) at protein levels by enzyme-linked immunosorbent assay (ELISA) with target-specific antibodies. Results showed both Tg(Ola.Sp7:N1aICD)^cy31^ and WT fish display similar expression levels for either cathepsin K (Figure 7M,O) or TRAP proteins (Figure 7N,P) aged at either embryonic stage (5 dpf) or young juvenile (35 dpf). We also validated the activities of several osteoblast- and osteoclast-regulated downstream genes by RT-PCR to confirm the activity of osteoblast and osteoclast at the mRNA level. Among several osteoblast-related marker genes tested, only *alp* (alkaline phosphatase) was significantly detected to upregulate at the mRNA level in Tg(Ola.Sp7:N1aICD)^cy31^. The other osteoblast-related makers such as *runx2a*, *runx2b*, *sp7*, *col1a1a* and *col1a1b* show no difference at the mRNA level (Figure 8A). For osteoclast markers, we found no significant difference of *rank*, *acp5b* and *ctsk* expression between WT and Tg(Ola.Sp7:N1aICD)^cy31^ (Figure 8B). It is also interesting to note that two genes correlated to bone remodeling, *opn* (osteopontin) and *phex* (phosphate-regulating neutral endopeptidase), were significantly downregulated in Tg(Ola.Sp7:N1aICD)^cy31^. Taken together, these observations support the idea that the hyperosteogeny in Tg(Ola.Sp7:N1aICD)^cy31^ is primarily caused by elevating osteoblast activity but not by promotion of osteoblast proliferation or inhibition of osteoclast activity (the proposed model was summarized in Figure 9). The deregulation of bone remodeling genes might lead to morphological change in the bone of Tg(Ola.Sp7:N1aICD)^cy31^.

## 3. Discussion

Notch signaling is an evolutionarily conserved mechanism for specifying and regulating organogenesis. Human and mouse genetic studies have demonstrated myriad mutations in the Notch signaling pathway that causes skeletal defects [19,20]. Previous studies showed conflicting results on the role of Notch signaling in bone homeostasis with either cell culture or mouse model. Stimulatory and inhibitory effects of Notch signaling pathway on osteogenesis were both reported [21,22,23]. Not only the calcified nodule formation was observed in a long-term culture of MC3T3-E1 osteoblastic cells with constitutive expression of Notch1 intracellular domain (NICD), but also the osteoblastic differentiation was promoted in the multipotent mesenchymal cell culture when the endogenous Notch1 was overexpressed. [21]. In addition, overexpressing NICD in primary human bone marrow mesenchymal stem cells (hMSCs) induced both spontaneous and stimulated osteoblastic cell differentiation [24].

Zebrafish has been well known as a great animal model for studying vertebrate development [25]. The benefits of applying zebrafish to study vertebrate biology, physiology, pathology, and toxicology are based on its high genomic conservation with mammals and rapid development and differentiation. Many advantages with zebrafish such as short life span, large number of offspring, low cost, and easy manipulation for generating transgenic as well as knock-out species, compared to mammalian models, have greatly sped up disease-based research in clinical studies [26,27,28,29,30,31,32]. We aimed to set up a zebrafish bone disease model and to clarify the role of Notch signaling in bone development using a zebrafish model. In this study, we overexpressed Notch1a intracellular domain (N1aICD) specifically in osteoblasts and found that the Tg(Ola.Sp7:N1aICD)^cy31^ transgenic fish showed the syndrome of hyperosteogeny with an increase of bone density, thicker spinal bones and narrower neural/hemal canals compared to WT. Moreover, we clearly demonstrated that hyperosteogeny phenotype in Tg(Ola.Sp7:N1aICD)^cy31^ fish is caused by elevating osteoblast activity instead of increasing osteoblast number or compromising osteoclast activity.

Generally, Notch has been suggested to be involved in the BMP-Smad1 pathway in osteoblastogenesis through Runx2 and Hey1 [23,32,33]. The osteosclerotic phenotype has been elucidated by the suppressive role of Notch-Rbpj signaling in osteoblastogenesis during the early stage of osteoblast differentiation [21,34]. Activation of Notch signaling in osteoblasts promotes cell proliferation and inhibits differentiation, leading to an osteosclerotic phenotype in transgenic mice [35]. Conditionally Cre-activated expression of NICD in osteoblasts caused massive osteosclerosis with growth retardation and abnormal vertebrae. Selective deletion of a Notch nuclear effector, Rbpj, completely suppressed the osteosclerotic and growth-retardation phenotypes in osteoblasts [21]. Notch RBPjk signaling functions in part through Hey1-mediated inhibition of NFATc1 to suppress osteoblastogenesis, contributing to bone homeostasis in vivo. In addition, the overexpression of Notch signaling has been reported to inhibit osteoblast differentiation [33,36] and mineralization [37]. In our study, we found that the overexpression of N1aICD in osteoblast can induce hyperosteogeny by promoting osteoblast differentiation and significantly elevating ALP activity. Taken together, our zebrafish model supports the idea that Notch overexpression in bone can promote the osteogenesis program [8,38]. It has been well-known that osteoblasts undergo three stages to develop into bone mass; cell proliferation, matrix maturation, and matrix mineralization [39]. During maturation and mineralization stages, alkaline phosphatase is maximally expressed and serves as a bone-specific marker [40]. Therefore, the augment of alkaline phosphatase activity is indeed a clue for us to deduce a model of hyperosteogeny, in which osteoblasts are activated through Notch signaling, and differentiate into osteocytes that become mineralized. In addition, we demonstrated the increased bone density may cause changes in locomotion of zebrafish (Figure 6D–I). The evidence that demonstrated a bone-related phenotype has been discussed. The tendon-ossified intramuscular bone in zebrafish showed lower swimming speed [41]. The difference between the model above and our N1aICD-transgenic fish was the ossified tendon which caused body stiffness, while increased bone density resulted in strengthening the body skeleton instead.

Bone remodeling is a process specified by a balance between bone formation by osteoblasts and bone resorption by osteoclasts [42]. An imbalance in bone remodeling contributes to several pathologic conditions, including osteosclerosis, osteopetrosis, and osteoporosis [43]. Osteosclerosis is a bone disorder characterized by an abnormal thickening and progressive increase in bone mass of the skeleton owing to an increased number of osteoblasts. In contrast, osteopetrosis results from a primary decrease in osteoclastic function [44]. Currently, extrinsic factors (i.e., fluorosis) that are associated with osteosclerosis have been reported [45]. However, reports of genetic factors or signaling pathways that are involved in osteosclerosis have been limited [21,46,47]. Among sclerosing-associated bone disease, myriad gene dysregulation patterns were examined [48]. According to the genetic validation in our hyperosteogency model, three genes showed significantly aberrant expression: *alp* was upregulated; *opn* and *phex* were downregulated. In both bone and calcifying cartilage, alkaline phosphatase (ALP) was expressed in early mineralization [49]. Consistent with the ALP and TRAP assay, *alp* expression was induced in a Notch-dependent manner. Accompanied by *vitamin D receptor a* (*vdra*) upregulation in our model, Ca^2+^ absorption in bone may be activated to support bone ossification. This is consistent with the finding of Vdra, but Vdrb is not important to Ca^2+^ homeostasis in zebrafish [50]. It has been reported that one of extracellular matrix (ECM)-targeted factors, Opn, when phosphorylated, inhibits mineralization in osteoblast in the mouse [51,52]. Moreover, Phex has been identified as a regulator that binds to Opn through an acidic serine- and aspartate-rich motif (ASARM) [53], which suggests dysregulation of Phex may fail to control osteogenesis along with Opn. Taken together, according to the gene expression pattern in our model, the lower expression of *opn* and *phex*, as well as higher expression of *alp* in Tg(Ola.Sp7:N1aICD)^cy31^ fish favor the differentiation (mineralization) of osteoblasts, leading to the hyperosteogeny.

In our zebrafish model, overexpressing of N1aICD specifically in osteoblasts induced high bone density and caused thicker spinal bones and narrower neural/hemal canals in a gender-specific manner. We hypothesized that different sex hormones might regulate Notch signaling in osteoblastic cells regarding osteoblastic differentiation. A loss-of-function of a Notch animal model may help in addressing gender-specific issue. However, identification of the factors contributing to such gender-specific difference is beyond the scope of the current study.

In summary, we established a hyperosteogeny model in zebrafish which may further our understanding of the osteogenesis-related signaling pathways. Our hyperosteogeny zebrafish model demonstrated that the mechanism of Notch induced bone disorder and may serve as a valuable platform to screen potential drugs for osteosclerosis therapy in the future.

## 4. Materials and Methods

### 4.1. Animal Ethics and Maintenance

All experimental protocols and procedures involving zebrafish were approved by the Committee for Animal Experimentation of the Chung Yuan Christian University (Number: CYCU9905, issue date 29 June 2010). All experiments were performed in accordance with the guidelines for laboratory animals. Fish were maintained as described in the zebrafish book [54]. The wild-type fish used was the AB strain. Fish were well fed with dry food and *Artemia salina* twice a day. The night before breeding, males and females were separated into different tanks at a ratio of one male to two females. At the beginning of the next light cycle, the males and females mate each other in the same tank. Embryos were collected and cultured in petri dishes containing E3 water (5.0 mM NaCl, 0.17 mM KCl, 0.33 mM CaCl_2_ and 0.33 mM MgSO_4_) at 28 °C under 14 h on/10 h off light cycle.

### 4.2. Plasmid Construction

Gateway *Tol2* system was used for generating overexpression clones [55]. For construction of 5′-end entry clone p5E-sp7, a DNA fragment representing medaka sp7 promoter region (5.3 kb) was generated from plasmid pmini-sp7-nlsGFP [56] by annealing the primer set 5′-TGAACATGTCAGTGCCATCA-3′ and 5′-ACTGGAGCCATAGCGAGTGTC-3′. The amplified fragment was cloned into pENTR™ 5′-TOPO^®^ vector (Life Technologies, Carlsbad, CA, USA) and transformed to DH5α competent cells. For the construction of a middle entry clone of pME-N1aICD (which continuously expresses active form of Notch1a), Notch1a intracellular domain fragment was generated from pCS2-MT-N1aICD by annealing the primer set 5′-GGGGACAAGTTTGTACAAAAAAGCAGGCTATGAACGAACCCAAAAAGAAGAGGAG-3′ and 5′-GGGGACCACTTTGTACAAGAAAGCTGGGTCTACTTGAAGGCTTCTGGAATATGG-3′ at *att*B1 and *att*B2 sites. The amplified fragment was processed via BP reaction with pDONR221 vector (Life Technologies). For construction of pDestTol2CG2-sp7-N1aICD-pA, p5E-sp7, pME-N1aICD and pDestTol2CG2 were combined to undergo LR reaction. The BP and LR reactions were carried out according to the protocol provided by Life Technologies, and the results were confirmed with PCR and the Sanger sequencing reaction. Approximately 2 µL of the construct (250 ng/μL) together with 2 µL of Transposase mRNA (250 ng/μL) and 6 µL of 0.5% phenol red were used to inject into ~400 freshly fertilized embryos. Since pDestTol2CG2 vector has a cmlc2-EGFP-pA reporter, the injected fish were raised to adulthood and screened for EGFP fluorescence in the heart region after 24 hpf (hour post fertilization). 

### 4.3. Genomic DNA Extraction

Injected fish were grown to adulthood and screened for stable transgenics. Caudal fin of adult fish was cut and heated in 100 µL of 50 mM NaOH at 95 °C for 20 min, and mixed with 20 μL of Tris-HCl neutralization solution. Excessive protein was precipitated by 100 μL of protein precipitation buffer (phenol:chloroform:isoamyl alcohol = 25:24:1) and then centrifuged at 13,000 rpm. Supernatant was mixed with one volume of isopropanol and centrifuged at 13,000 rpm. Pure genomic DNA pellet was washed with 70% ethanol twice and dissolved in DNase and RNase-free water. The purified genomic DNA can be stored at −20 °C for up to 6 months.

### 4.4. Calcein Labeling for Bone Formation Quantification

Calcein is a vital fluorescent dye used for detecting calcium as an index of bone mineralization [57]. Zebrafish embryos aged at 6 to 7 dpf were immersed in 1% Calcein solution (C0875, Sigma-Aldrich, St. Louis, MO, USA) for 5 min and washed with E3 water three times to remove unbound dye. Quantification of bone calcification was performed according to our published method [58].

### 4.5. Bone and Cartilage Dual Staining

Alcian blue and Alizarin red double stains were performed to label cartilage and bone by following the previous method [59]. Alcian blue stains acid mucosubstance, acetic mucin, and sulfated as well as carboxylated mucopolysaccharides in developing cartilage. Zebrafish were fixed with 4% paraformaldehyde (PFA)/PBS for 16 h at 4 °C, rinsed with water for a few minutes and stained in 0.1 mg/mL Alcian blue solution (D0026, GeneMarkBio, Taipei, Taiwan) for another overnight. Zebrafish were rehydrated for 30 min with ethanol/H_2_O series (95, 70, 40, and 15% ethanol) and rinsed with water for 30 min for the final step. At last, the specimens were soaked in 20 mg/mL trypsin in saturated sodium tetraborate for 3 h at room temperature to dissolve unwanted soft tissue. Excessive dye was removed with 1% KOH/3% H_2_O_2_ (*v*/*v*) until conformation of cartilage was fully observed. As for vertebrate skeletal observation, alizarin red was used. The specimens were then stained with 1 mg/mL Alizarin red (AD0144, GeneMarkBio, Taipei, Taiwan) in 1% (*w*/*w*) KOH at room temperature overnight. Finally, the zebrafish were immersed in 1% KOH/glycerol with shaking to remove excessive dye. Specimens were stored in 100% glycerol for future usage.

### 4.6. Whole-Mount Immunostaining

Zebrafish were anesthetized with 0.16% Tricaine and scales were collected. Scales were fixed in 4% PFA for 12 h at 4 °C. After an extensive wash in PBS, scales were blocked with 3% BSA/PBST (Phosphate-buffered saline, Tween-20 in 1% (*v*/*v*) solution) at room temperature for 60 min, and incubated with 1:200 diluted primary antibodies (anti-phospho-Histone 3 Ser-10, Santa Cruz, sc-8656-R) overnight. After an extensive wash in PBST, scales were incubated with 1:500 diluted Goat-anti-rabbit Alexa Fluor 488-conjugated secondary antibodies (Molecular probe, A11008) for fluorescent signals.

### 4.7. Histology

Plastic section was performed for histological analysis of bone tissue of zebrafish. Tissue was fixed overnight in 4% PFA at 4 °C and then dehydrated overnight in 50, 75, and 100% ethanol. After complete dehydration, samples were infiltrated and embedded in Technovit 7100 resin (HeraeusKulzer, Deutschland, German). Samples were sectioned at 2 μm intervals and stained with Toluidine blue staining.

### 4.8. ALP Staining and ALP Activity Detection

It is known that alkaline phosphatase (ALP) activity is essential for osteoblasts. Scales of adult zebrafish were collected and subjected to ALP staining in a BCIP/NBT liquid substrate system (Sigma, B1911). After a rinse with water, samples were then stained with acid hematoxylin for 5 min. Quantitative detection of ALP activity was performed using pNPP (p-nitro-phenyl phosphate) as a substrate by following the previous method [16]. Scales were fixed with 4% PFA in 96-well plate overnight. After removal of 4% PFA, alkaline buffer (100 mM Tri-HCl, pH 9.5, 1 mM MgCl_2_, 0.1 mM ZnCl_2_) was added and incubated for 30 min. Alkaline buffer was replaced with 20 mM pNPP/alkaline buffer and incubation continued for another 1 h. Finally, 2N NaOH was applied for stopping reaction, and absorbance was measured by an ELISA plate reader (Multiskan GO, Thermo Fisher Scientific, Waltham, MA, USA) at 405 nm.

### 4.9. Detection of Cathepsin K and TRAP Expression by ELISA

A pool of 30 zebrafish embryos was used to prepare an independent homogenate sample, which was homogenized on ice in 50 volumes (*v*/*w*) of phosphate-buffered saline (PBS) at pH 7.2. Samples were then centrifuged at 15,000 rpm for 15 min at 4 °C, and the supernatant was kept in microtubes at −80 °C for further assays. Total protein concentration was determined using a Pierce BCA Protein Assay Kit (23225, Thermo Fisher Scientific, Waltham, MA, USA). The color formation was analyzed at 562 nm using a microplate reader (Multiskan GO, Thermo Fisher Scientific, Waltham, MA, USA). Later, ELISA kits were used to measure the relative concentration of cathepsin K and TRAP in whole embryo lysates according to the manufacturer’s instructions (ZG-E1640 and ZG-E1641, Zgenebio Company, Taipei, Taiwan). The color change was measured spectrophotometrically at a wavelength of 450 nm using a microplate reader (Multiskan GO, Thermo Fisher Scientific, Waltham, MA, USA).

### 4.10. Micro CT

Adult zebrafish were fixed in 4% PFA overnight, washed with PBST, and embedded in 1% agarose gel in a 2.0 mL tube. Vertebral topography was imaged in a SkyScan 1174 micro-CT scanner (Bruker, Kontich, Belgium) using a 6 μm spatial resolution. The applied x-ray voltage was 50 kV, and scans were over 180° with a 0.3° rotation step. Images were reconstructed and binarized with global threshold using SkyScan CTAn software, as described. A region of interest was traced around individual vertebrae and surface-rendered models prepared using the “Double Time Cubes” 3D reconstruction method. Cortical bone mineral density (BMD) was estimated by comparing bone density with calibration phantoms of known BMD, scanned at the same time as the vertebrae.

### 4.11. Morphometric Analysis

The morphology of adult zebrafish was recorded by digital camera (Canon EOS 600D, Tokyo, Japan). The images were converted into tps format using TpsUtil software and digitized by using TpsDig2 software (http://life.bio.sunysb.edu/morph/soft-utility.html). Later, Morpho J [60] was used to generate covariance matrix and perform procrustes analysis as well as principal component analysis (PCA) with default settings.

### 4.12. Locomotion Activity Assay

We performed 3D swimming activity assay for comparing the locomotion activity between WT and transgenic fish. The instrumental setup and protocol for 3D swimming tracking are based on our previously published method [15].

### 4.13. Quantitative Real-Time PCR

Total RNA from zebrafish embryos or tissue were harvested with the RNAZol^®^RT (Life Technologies, Carlsbad, CA, USA) and quantified with NanoDrop (Thermo Scientific, Madison, WI, USA). RevertAid first cDNA synthesis kit (K1622, Thermo Scientific, Waltham, MA, USA) was used to synthesize first-strand cDNA from total zebrafish RNA according to the manufacturer’s instructions. Quantitative real-time PCR (qPCR) was performed using iQ SYBR Green Supermix (Bio-Rad Laboratories, Hercules, CA, USA) on a Bio-Rad iCycler using *β-actin* as control, and data were analyzed using the ∆∆*C*t method [61]. All primer sequences used in this study are listed in Table 1.

### 4.14. Statistical Analysis

The data were expressed as mean ± SD and tested by Student’s *t* test. *p* < 0.05 was identified to be statistically significant.

## Figures and Tables

**Table 1:** Primer sets for Real Time-PCR.

Gene	Forward (5′ to 3′)	Reverse (5′ to 3′)
**Osteoblast Differentiation Makers**		
*runx2a*	GACGGTGGTGACGGTAATGG	TGCGGTGGGTTCGTGAATA
*runx2b*	CGGCTCCTACCAGTTCTCCA	CCATCTCCCTCCACTCCTCC
*sp7 (osterix)*	GGCTATGCTAACTGCGACCTG	GCTTTCATTGCGTCCGTTTT
*col1a1a*	GAATAAGCAGGTGGAGTCT	GAAGCAACTGAACGACATT
*col1a1b*	GGTGCTATGCTGTGATTCT	GATTCTCGCTAAGTGTCCAT
*alp*	CAAGAACTCAACAAGAAC	TGAGCATTGGTGTTATAC
*osteopontin*	GCCTCCATCATCATCGTA	AATCACCAAGCACCAGTA
*phex*	GAGAATGAATGGATGGATGA	TTGATGTCTTCGTTAATATAGGT
**Osteoclast Differentiation Markers**		
*ctsk*	GGACTCAATCACTATCACT	AGAACAAGACATCTAAGACA
*rank*	GCACGGTTATTGTTGTTA	TATTCAGAGGTGGTGTTAT
*acp5b*	GCTGCTGCTAACAAACAAT	GACCAACCACGATGACAA

## References

[1] Lindsell C.E., Boulter J., DiSibio G., Gossler A., Weinmaster G. (1996). Expression Patterns of Jagged, Delta1, Notch1, Notch2, and Notch3 Genes Identify Ligand–Receptor Pairs That May Function in Neural Development. Mol. Cell. Neurosci..

[2] Eiraku M., Hirata Y., Takeshima H., Hirano T., Kengaku M. (2002). Delta/Notch-like Epidermal Growth Factor (EGF)-related Receptor, a Novel EGF-like Repeat-containing Protein Targeted to Dendrites of Developing and Adult Central Nervous System Neurons. J. Boil. Chem..

[3] Hu Q.-D., Ang B.-T., Karsak M., Hu W.-P., Cui X.-Y., Duka T., Takeda Y., Chia W., Sankar N., Ng Y.-K. (2003). F3/Contactin Acts as a Functional Ligand for Notch during Oligodendrocyte Maturation. Cell.

[4] Kopan R., Ilagan M.X.G. (2009). The Canonical Notch Signaling Pathway: Unfolding the Activation Mechanism. Cell.

[5] Groot A.J., Vooijs M.A. (2012). The Role of Adams in Notch Signaling. Results Probl. Cell Differ..

[6] D’Souza B., Meloty-Kapella L., Weinmaster G. (2010). Canonical and Non-Canonical Notch Ligands.

[7] Zhang H., Hilton M.J., Anolik J.H., Welle S.L., Zhao C., Yao Z., Li X., Wang Z., Boyce B.F., Xing L. (2014). Notch inhibits osteoblast formation in inflammatory arthritis via noncanonical nf-kappab. J. Clin. Investig..

[8] Engin F., Yao Z., Yang T., Zhou G., Bertin T., Jiang M.M., Chen Y., Wang L., Zheng H., Sutton R.E. (2008). Dimorphic effects of Notch signaling in bone homeostasis. Nat. Med..

[9] Zanotti S., Smerdel-Ramoya A., Stadmeyer L., Durant D., Radtke F., Canalis E. (2008). Notch Inhibits Osteoblast Differentiation and Causes Osteopenia. Endocrinology.

[10] Ashley J.W., Ahn J., Hankenson K.D. (2015). Notch signaling promotes osteoclast maturation and resorptive activity. J. Cell. Biochem..

[11] Bai S., Kopan R., Zou W., Hilton M.J., Ong C.T., Long F., Ross F.P., Teitelbaum S.L. (2008). Notch1 regulates osteoclastogenesis directly in osteoclast precursors and indirectly via osteoblast lineage cells. J. Biol. Chem..

[12] Zanotti S., Canalis E. (2010). Notch and the skeleton. Mol. Cell. Biol..

[13] Fukushima H., Nakao A., Okamoto F., Shin M., Kajiya H., Sakano S., Bigas A., Jimi E., Okabe K. (2008). The association of notch2 and nf-kappab accelerates rankl-induced osteoclastogenesis. Mol. Cell. Biol..

[14] DeLaurier A., Eames B.F., Blanco-Sánchez B., Peng G., He X., Swartz M.E., Ullmann B., Westerfield M., Kimmel C.B., Blanco-Sánchez B. (2010). Zebrafish sp.7:EGFP: A transgenic for studying otic vesicle formation, skeletogenesis, and bone regeneration. Genesis.

[15] Audira G., Sampurna B.P., Juniardi S., Liang S.-T., Lai Y.-H., Hsiao C.-D. (2018). A Simple Setup to Perform 3D Locomotion Tracking in Zebrafish by Using a Single Camera. Inventions.

[16] Pasqualetti S., Banfi G., Mariotti M. (2012). Osteoblast and osteoclast behavior in Zebrafish cultured scales. Cell Tissue Res..

[17] Takahashi H., Suzuki N., Takagi C., Ikegame M., Yamamoto T., Takahashi A., Moriyama S., Hattori A., Sakamoto T. (2008). Prolactin Inhibits Osteoclastic Activity in the Goldfish Scale: A Novel Direct Action of Prolactin in Teleosts. Zoölog. Sci..

[18] Suzuki N., Sato M., Nassar H.F., Abdel-Gawad F.K., Bassem S.M., Yachiguchi K., Tabuchi Y., Endo M., Sekiguchi T., Urata M. (2016). Seawater Polluted with Highly Concentrated Polycyclic Aromatic Hydrocarbons Suppresses Osteoblastic Activity in the Scales of Goldfish, Carassius auratus. Zoölog. Sci..

[19] Bulman M.P., Kusumi K., Frayling T.M., McKeown C., Garrett C., Lander E.S., Krumlauf R., Hattersley A.T., Ellard S., Turnpenny P.D. (2000). Mutations in the human Delta homologue, DLL3, cause axial skeletal defects in spondylocostal dysostosis. Nat. Genet..

[20] Dunwoodie S.L., Clements M., Sparrow D.B., Sa X., Conlon R.A., Beddington R.S.P. (2002). Axial skeletal defects caused by mutation in the spondylocostal dysplasia/pudgy gene Dll3 are associated with disruption of the segmentation clock within the presomitic mesoderm. Development.

[21] Hilton M.J., Tu X., Wu X., Bai S., Zhao H., Kobayashi T., Kronenberg H.M., Teitelbaum S.L., Ross F.P., Kopan R. (2008). Notch signaling maintains bone marrow mesenchymal progenitors by suppressing osteoblast differentiation. Nat. Med..

[22] Tezuka K.-I., Yasuda M., Watanabe N., Morimura N., Kuroda K., Miyatani S., Hozumi N. (2002). Stimulation of Osteoblastic Cell Differentiation by Notch. J. Bone Miner. Res..

[23] Engin F., Lee B. (2010). Notching the bone: Insights into multi-functionality. Bone.

[24] Lu Z. (2004). Notch signaling stimulates osteogenic differentiation of human bone marrow-derived mesenchymal stem cells. Chin. Sci. Bull..

[25] Hoo J.Y., Kumari Y., Shaikh M.F., Hue S.M., Goh B.H. (2016). *Zebrafish*: A Versatile Animal Model for Fertility Research. BioMed Res. Int..

[26] Lieschke G.J., Currie P.D. (2007). Animal models of human disease: *Zebrafish* swim into view. Nat. Rev. Genet..

[27] Dooley K., Zon L.I. (2000). Zebrafish: A model system for the study of human disease. Curr. Opin. Genet. Dev..

[28] Kari G., Rodeck U., Dicker A.P., Dicker A. (2007). Zebrafish: An Emerging Model System for Human Disease and Drug Discovery. Clin. Pharmacol. Ther..

[29] Santoriello C., Zon L.I. (2012). Hooked! Modeling human disease in Zebrafish. J. Clin. Investig..

[30] Howe K., Clark M.D., Torroja C.F., Torrance J., Berthelot C., Muffato M., Collins J.E., Humphray S., McLaren K., Matthews L. (2013). The Zebrafish reference genome sequence and its relationship to the human genome. Nature.

[31] Barut B.A., Zon L.I. (2000). Realizing the potential of Zebrafish as a model for human disease. Physiol. Genom..

[32] Phillips J.B., Westerfield M. (2014). Zebrafish models in translational research: Tipping the scales toward advancements in human health. Dis. Model. Mech..

[33] Zhu F., Friedman M.S., Luo W., Woolf P., Hankenson K.D. (2012). The transcription factor osterix (sp7) regulates bmp6-induced human osteoblast differentiation. J. Cell. Physiol..

[34] Zamurovic N., Cappellen D., Rohner D., Susa M. (2004). Coordinated Activation of Notch, Wnt, and Transforming Growth Factor-β Signaling Pathways in Bone Morphogenic Protein 2-induced Osteogenesis. J. Boil. Chem..

[35] Tao J., Chen S., Yang T., Dawson B., Munivez E., Bertin T., Lee B. (2010). Osteosclerosis owing to notch gain of function is solely rbpj-dependent. J. Bone Miner. Res..

[36] Tu X., Chen J., Lim J., Karner C.M., Lee S.Y., Heisig J., Wiese C., Surendran K., Kopan R., Gessler M. (2012). Physiological notch signaling maintains bone homeostasis via rbpjk and hey upstream of nfatc1. PLoS Genet..

[37] Canalis E., Bridgewater D., Schilling L., Zanotti S. (2016). Canonical notch activation in osteocytes causes osteopetrosis. Am. J. Physiol. Endocrinol. Metab..

[38] Sciaudone M., Gazzerro E., Priest L., Delany A.M., Canalis E. (2003). Notch 1 Impairs Osteoblastic Cell Differentiation. Endocrinology.

[39] Shao J., Zhou Y., Lin J., Nguyen T.D., Huang R., Gu Y., Friis T., Crawford R., Xiao Y. (2018). Notch expressed by osteocytes plays a critical role in mineralisation. J. Mol. Med..

[40] Ji Y., Ke Y., Gao S. (2017). Intermittent activation of notch signaling promotes bone formation. Am. J. Transl. Res..

[41] Stein G.S., Lian J.B. (1993). Molecular Mechanisms Mediating Proliferation/Differentiation Interrelationships during Progressive Development of the Osteoblast Phenotype. Endocr. Rev..

[42] Golub E., Harrison G., Taylor A., Camper S., Shapiro I. (1992). The role of alkaline phosphatase in cartilage mineralization. Bone Miner..

[43] Yao W., Lv Y., Gong X., Wu J., Bao B. (2015). Different ossification patterns of intermuscular bones in fish with different swimming modes. Boil. Open.

[44] Crockett J.C., Rogers M.J., Coxon F.P., Hocking L.J., Helfrich M.H. (2011). Bone remodelling at a glance. J. Cell Sci..

[45] Feng X., McDonald J.M. (2011). Disorders of bone remodeling. Annu. Rev. Pathol..

[46] Gowen M., Pavasovic D., Lazner F., Kola I. (1999). Osteopetrosis and osteoporosis: Two sides of the same coin. Hum. Mol. Genet..

[47] Wiener R.C., Sambamoorthi U. (2013). Dental fluorosis and lumbar spine bone mineral density in adults, ages 20 to 49 years: Results from the 2003 to 2004 National Health and Nutrition Examination Survey. J. Dent. Hyg. JDH.

[48] Jochum W., David J.-P., Elliott C., Wutz A., Plenk H., Matsuo K., Wagner E.F. (2000). Increased bone formation and osteosclerosis in mice overexpressing the transcription factor Fra-1. Nat. Med..

[49] Ihde L.L., Forrester D.M., Gottsegen C., Masih S., Patel D.B., Vachon L.A., White E.A., Matcuk G.R. (2011). Sclerosing Bone Dysplasias: Review and Differentiation from Other Causes of Osteosclerosis. Radiographics.

[50] De Vernejoul M.C. (2008). Sclerosing bone disorders. Best Pract. Res. Clin. Rheumatol..

[51] Hoemann C., EI-Gabalawy H., McKee M., Hoemann C. (2009). In vitro osteogenesis assays: Influence of the primary cell source on alkaline phosphatase activity and mineralization. Pathol. Boil..

[52] Lin C.H., Su C.H., Tseng D.Y., Ding F.C., Hwang P.P. (2012). Action of vitamin d and the receptor, vdra, in calcium handling in Zebrafish (*Danio rerio*). PLoS ONE.

[53] Narisawa S., Yadav M.C., Millán J.L. (2013). In Vivo Overexpression of Tissue-Nonspecific Alkaline Phosphatase Increases Skeletal Mineralization and Affects the Phosphorylation Status of Osteopontin. J. Bone Miner. Res..

[54] Westerfield M. (1995). The Zebrafish Book: A Guide for the Laboratory Use of Zebrafish (Brachydanio Rerio).

[55] Kwan K.M., Fujimoto E., Grabher C., Mangum B.D., Hardy M.E., Campbell D.S., Parant J.M., Yost H.J., Kanki J.P., Chien C.-B. (2007). The Tol2kit: A multisite gateway-based construction kit for Tol2 transposon transgenesis constructs. Dev. Dyn..

[56] Spoorendonk K.M., Peterson-Maduro J., Renn J., Trowe T., Kranenbarg S., Winkler C., Schulte-Merker S. (2008). Retinoic acid and Cyp26b1 are critical regulators of osteogenesis in the axial skeleton. Development.

[57] Du S.J., Frenkel V., Kindschi G., Zohar Y. (2001). Visualizing Normal and Defective Bone Development in *Zebrafish* Embryos Using the Fluorescent Chromophore Calcein. Dev. Boil..

[58] Chen J.-R., Lai Y.-H., Tsai J.-J., Hsiao C.-D. (2017). Live Florescent Staining Platform Drug-Screening and Mechanism-Analysis in Zebrafish for Bone Mineralization. Life Sci..

[59] Kelly W.L., Bryden M.M. (1983). A Modified Differential Stain for Cartilage and Bone in Whole Mount Preparations of Mammalian Fetuses and Small Vertebrates. Stain. Technol..

[60] Klingenberg C.P. (2011). Morphoj: An integrated software package for geometric morphometrics. Mol. Ecol. Resour..

[61] Schmittgen T.D., Livak K.J. (2008). Analyzing real-time PCR data by the comparative CT method. Nat. Protoc..

